# Susceptibility and severity of Covid-19 in different blood groups

**DOI:** 10.6026/973206300211581

**Published:** 2025-06-30

**Authors:** Ritesh Upadhyay, Ravikant Mahale, Pawan Nandurkar, Sonali Tripathi

**Affiliations:** 1 Department of Community Medicine, Chhindwara Institute of Medical Sciences, Mandhata, Shivam Sundaram Colony, Chhindwara, Madhya Pradesh 480001, Madhya Pradesh, India; 2 Department of Pathology, Chhindwara Institute of Medical Sciences, Mandhata, Shivam Sundaram Colony, Chhindwara, Madhya Pradesh 480001, Madhya Pradesh, India; 3 Department of Pediatrics, Chhindwara Institute of Medical Sciences, Mandhata, Shivam Sundaram Colony, Chhindwara, Madhya Pradesh 480001, India; 4 Department of Anesthesiology, Chhindwara Institute of Medical Sciences, Mandhata, Shivam Sundaram Colony, Chhindwara, Madhya Pradesh 480001, Madhya Pradesh, India

**Keywords:** COVID-19, ABO blood group, Rh factor, susceptibility, severity, observational study

## Abstract

The relation of ABO and Rh blood groups with susceptibility and severity of COVID-19 among 120 patients is it of interest. Blood group A showed the highest infection rate of 37.5%, while the lowest was that of blood group O, at 20%. Susceptibility was a little higher in Rh-positive patients and the highest prevalence of severe disease was found among patients with blood group AB, at 30%. Results show that groups A and AB are associated with increased susceptibility and severity, which may help identify at-risk populations to target public health interventions. Further studies can help refine such associations and inform preventive strategies.

## Background:

The novel coronavirus disease 2019 (COVID-19) caused by the virus SARS-CoV-2, a lot of effort has been given to seek factors that influence susceptibility and severity of the infection. Among these factors are the ABO and Rh blood group systems for their potential role in infectious diseases [1]. It has been observed by several earlier researchers that blood group antigens could play a role in susceptibility to certain diseases by acting as receptors or co-receptors [2]. Some early reports from Wuhan, China, suggested that there was an association between the susceptibility to COVID-19 and the ABO blood group, with those with the blood group A being more susceptible and those with group O showing some degree of protection [3]. The subsequent reports are mixed, though and similar patterns have been observed in different populations [4, 5]. These associations remain under investigation and may be attributed to natural antibodies and the part played by antigens of the blood group in immune responses [6]. Another important determinant in blood grouping is the Rh factor, with its D antigen. However, very few studies have evaluated the relationship between Rh factor and COVID-19 and results are not very conclusive [7]. Further studies on potential associations between Rh factor and COVID-19 may shed more light on disease mechanisms. With the pervasiveness of COVID-19 and the call for proper risk stratification, the present study has been conducted to find the association between ABO and Rh blood groups with susceptibility and severity of COVID-19 within a diverse population. We attempt to add to this growing body of evidence by reviewing a large database from a regional healthcare system to help build targeted public health approaches [8]. Therefore, it is of interest to describe the relation of ABO and Rh blood groups with susceptibility and severity of COVID-19 among 120 patients.

## Materials and Methods:

A retrospective observational study was conducted from January 2021 to January 2022 at the Chhindwara Institute of Medical Sciences, Chhindwara (MP) Hospital after taking approval from the institutional ethics committee (Ref. No. CIMS/EC/2022/635). The study population consisted of 120 patients according to the strict eligibility criteria used for the participants. The main criteria for their inclusion were a documented ABO and Rh blood group information with confirmed COVID-19 RT-PCR test results. Exclusion criteria included incomplete medical records and lack of information about blood groups. Data collection was done by obtaining demographic information such as age, sex and ethnicity. ABO and Rh factor statuses of the patients were obtained from hospital records. COVID-19 status was confirmed by RT-PCR testing. The severity of the disease was classified into three categories: mild cases (managed at home), moderate cases (requiring hospital admission) and severe cases (necessitating intensive care or mechanical ventilation). Statistical analysis was conducted to analyze the data gathered. Descriptive statistics were used to summarize patient demographics and the distribution of blood groups. The chi-square test was used to determine the association between blood groups and COVID-19 infection rates. Logistic regression analysis was used to control for confounding variables such as age and sex. Statistical significance was determined at a p-value of less than 0.05. All analyses were conducted using SPSS software, version 26.0.

## Results:

The tables presented in the research give insight into the connection of blood types with COVID-19. It is noted, from Table 1 (see PDF), that the ABO and Rh blood groups were prevalent and, among them, blood group A and O emerged as the majority. From Table 2 (see PDF), it is derived that blood type A had a maximum COVID positivity rate, whereas group O emerged as the smallest. Table 3 (see PDF) analyzes COVID-19 positivity with respect to the Rh factor. No statistically significant differences between the Rh-positive and Rh-negative population were observed. Table 4 (see PDF) reports on disease severity according to blood group, revealing a greater number of severe cases in blood group AB. Collectively, these findings point out that A and AB blood groups are associated with increased susceptibility and severity while blood group O seems to be protective, though Rh factor was not significant. Out of 120 patients, the distribution of ABO and Rh blood groups is presented in Table 1 (see PDF). Among the 120 patients, 48 tested positive for COVID-19. The distribution of positive cases by blood group is shown in Table 2 (see PDF). The prevalence of COVID-19 positive cases is presented regarding the proportion of blood groups. The positivity is found to be maximum in blood group A and minimum in blood group O. Statistical analysis indicated that individuals with blood group A had a higher risk of contracting COVID-19 compared to blood group O (p=0.04) as shown in Figure 1 (see PDF). The distribution of COVID-19 positivity concerning Rh factor is shown in Table 3 (see PDF). There was no statistically significant difference in COVID-19 infection rates between Rh-positive and Rh-negative individuals (p=0.75). Distribution of severity in COVID-19 positive patients, which reveals that more patients have been severe with blood group AB as compared to others. These results indicate a link between group A blood type with higher susceptibility and blood type AB with greater severity of COVID-19; in contrast, the O group is associated with lower susceptibility and no cases of severe COVID-19. Patients with blood group AB had a higher proportion of severe cases (25%) compared to other groups as shown in Figure 2 (see PDF). Among the 48 COVID-19 positive patients, disease severity by blood group is detailed in Table 4 (see PDF).

## Discussion:

This study established a correlation of ABO and Rh blood groups with susceptibility to COVID-19 as well as severity in 120 patients. From the obtained results, patients having blood group A are likely to contract the virus than the patient with the "O" blood group; this correlates with previous findings by other scholars [9]. Potential mechanisms thought to explain this association relate to the blood group antigens function in viral entry and the response to immunity [10]. An antigen may act to favor attachment of SARS-CoV-2 to the host cells that lead to higher susceptibility [11]. Natural antibodies to anti-A and anti-B may confer a protective effect for the O phenotype because of virus neutralization [12]. It showed no significant association between Rh factor and COVID-19 infection rates; this is supported by other studies that show that the Rh factor may not play a critical role in susceptibility [13]. However, the number of samples obtained for Rh-negative individuals was few and thus further studies using larger cohorts are required [14]. In terms of disease severity, blood group AB patients had a higher proportion of severe cases. This observation indicates that AB blood group individuals may have an abnormal response to the immune system and the results would be worse in comparison [15, 16]. As there are few patients of the AB group, this result needs to be viewed with some caution [17]. This study has a few limitations that would have implications on the interpretation of the results. Firstly, its sample size was small and in subgroups, for blood group, thereby diminishing statistical power and the strength of conclusions. Second, by virtue of the retrospective nature of this study, it might also have problems of selection bias and the presence of confounding variables not considered in the analysis. In the last point, since the data were gathered from a single hospital, generalizing the findings might be difficult toward broader or diversified populations. Hence, the readers should take such factors into consideration while interpreting the results of this study. These associations should be confirmed by more extensive, multicenter studies that could shed more light on the underlying biological mechanisms. Elucidating how blood groups affect COVID-19 could allow for improved risk stratification and inform prevention strategies with targets.

## Conclusion:

Individuals with blood group A are more prone to COVID-19 and those with blood group O may be less prone. Blood group AB may be linked with increased disease severity. However, there was no association found with the Rh factor. These findings are part of the growing evidence about the role of blood groups in COVID-19 and may be helpful in identifying high-risk populations for targeted interventions.
